# Why solutions to antimicrobial resistance are urgently needed

**DOI:** 10.1242/dmm.052789

**Published:** 2025-12-19

**Authors:** Serge Mostowy, Katherine R. Duncan

**Affiliations:** ^1^ Department of Infection Biology, London School of Hygiene & Tropical Medicine, London WC1E 7HT, UK; ^2^ Biosciences Institute, Newcastle University, Newcastle upon Tyne NE2 4HH, UK

## Abstract

**Summary:** This Editorial discusses the growing global problem of antimicrobial resistance, and new and emerging opportunities for developing real-world solutions, involving both basic science and clinical applications.

Antimicrobial resistance (AMR) is a major global threat that concerns both emerging and existing infections. AMR occurs when microorganisms, like bacteria, viruses, fungi and parasites, evolve to resist the medicines designed to kill them, such as antibacterials, antivirals and antifungals (see also Sanyal, 2023). When the resulting ‘superbugs’ cause common infections, they become difficult or impossible to treat, threatening the effectiveness of modern medicine. What is particularly concerning is the increasing frequency of multidrug-resistant (MDR) strains and extensively drug-resistant (XDR) strains, as even last-line antibiotics are being rendered ineffective. AMR is associated with significant morbidity and mortality – deaths linked to antibiotic-resistant superbugs increased by 17% in England in 2024 (data from the UK Health Security Agency, November 2025). The Global Research on Antimicrobial Resistance (GRAM) project suggests that, if no steps are taken, 39 million deaths will be directly attributable to bacterial AMR between 2025 and 2050, which equates to approximately three deaths every minute. Treating AMR infections is a significant economic burden, costing the National Health Service in England about £180 million a year. Alarmingly, the World Bank's modelling data suggest that, by 2030, AMR could lead to GDP losses of US$ 1.0 to 3.4 trillion per year.

To mitigate this threat, a global ‘One Health’ approach is crucial: integrating human and animal health, agriculture and the environment. It is also vital to improve public awareness and knowledge, strengthen surveillance, optimise antimicrobial stewardship, prevent infections and transmission of resistant organisms, fund research into new treatments, and improve the governance of and policies supporting drug development. To showcase these issues, Disease Models & Mechanisms (DMM) hosted a one-day symposium as part of the 100-year anniversary conference, organized by the publisher of DMM – The Company of Biologists (see Fig. 1). Entitled ‘Interdisciplinary approaches to combatting antimicrobial resistance’, the event was organised by Katherine Duncan (Newcastle University, UK) and DMM Editorial Advisory Board member Serge Mostowy (London School of Hygiene & Tropical Medicine, UK). They invited international experts in the field, who are at different stages of their own research careers and use a breadth of approaches to combat AMR, ranging from fundamental basic science discovery to clinical research and real-world impact. With this symposium, our goal was to draw attention to exciting and emerging concepts in AMR, and to discuss real-world solutions. Finally, we hoped to introduce DMM readers to the multifaceted disciplines underlying AMR science and inspire ways in which their own research may one day contribute.

**Fig. 1.**
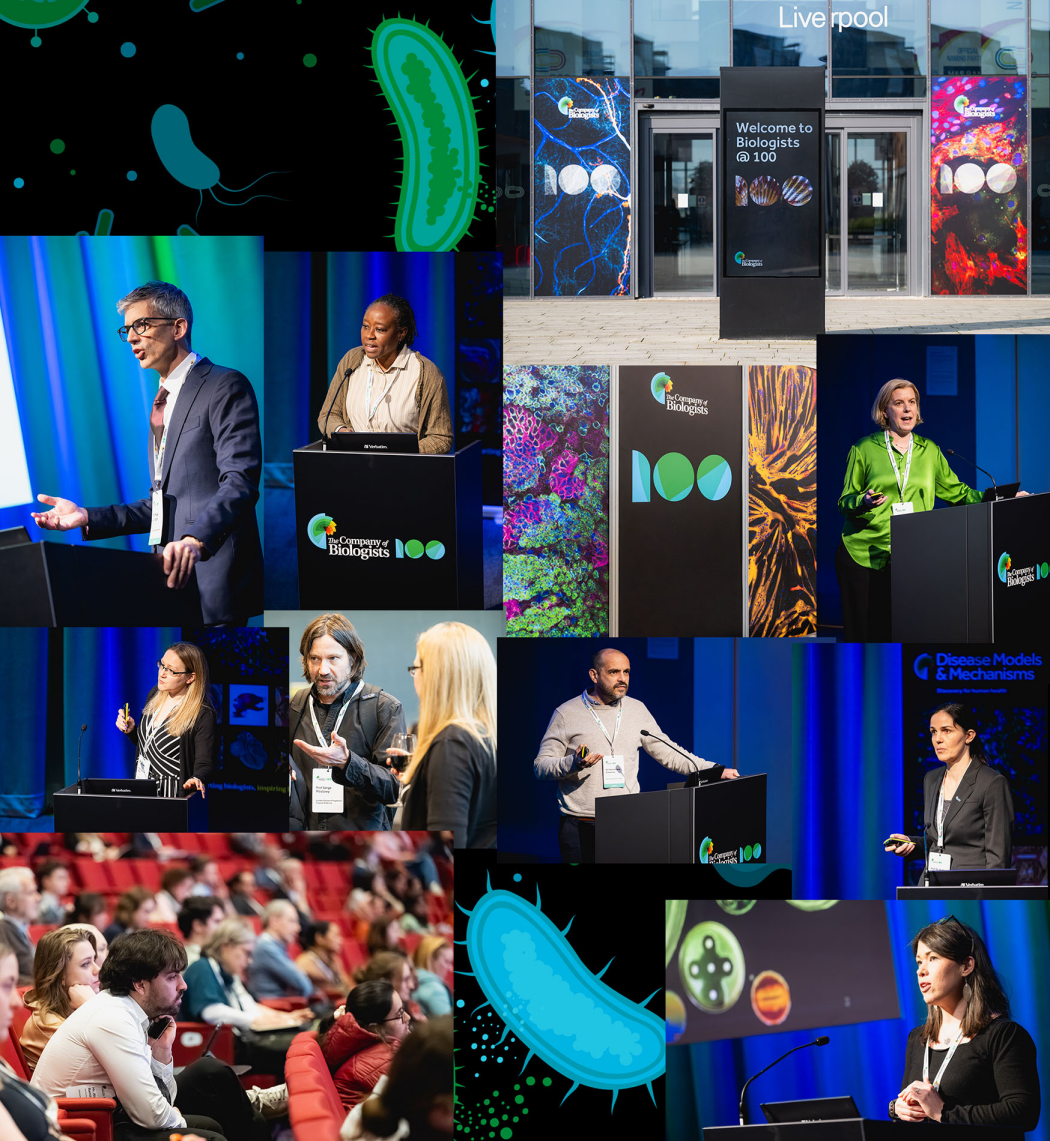
Images from the ‘Interdisciplinary approaches to combatting antimicrobial resistance’ symposium, part of the 100-year anniversary conference organized by The Company of Biologists.

## Drug discovery and ‘omics

Natural products (arising from living organism chemistry) have long been a source of medicine (Mostowy, 2022). Now, with modern ‘omics approaches, drug discovery is in a new era, with data-informed biodiscovery being central to tackling AMR. To this end, **Roger Linington** (Simon Fraser University, Canada) works at the interface of chemistry and biology. He uses innovative approaches to understand microbial natural products, including developing high-throughput screening platforms for functional annotation of molecules. He also links mass spectrometry and nuclear magnetic resonance-based metabolomics platforms to antibiotic screening data. Roger presented the global gold-standard database of all published microbial natural products – the Natural Products Atlas. The latest version contains over 36,000 molecules, allows chemical space to be mapped by chemical architecture and source organism, and facilitates the linking of biosynthetic genes to molecules. This enables a global understanding of where interesting chemistry exists and pinpoints chemical novelty, information urgently needed to tackle AMR.

**Nadine Ziemert** (University of Tübingen, Germany) pioneers bioinformatic tools for natural product biodiscovery and harnesses the power of predictive computational work to inform biodiscovery efforts. Her work has highlighted the gap between the potential of bacteria to produce antibiotics and our ability to see that chemistry in the laboratory, with a particular focus on the genomes of understudied bacterial genera with high antimicrobial potential. Nadine is also pioneering state-of-the-art methods for understanding bacterial resistance, such as the Antibiotic Resistant Target Seeker (ARTS). This enables researchers to computationally detect and predict biosynthetic gene clusters and their mode of action. It has also recently been developed to assess fungal genomes. Her work encapsulates the predictive power of genetic information to prioritize strains, genes and targets of interest. This is greatly needed to ensure that researchers focus on promising strains and molecules.

## Cellular mechanisms of AMR

**Jessica Blair** (University of Birmingham, UK) aims to understand the molecular mechanisms behind how bacteria become resistant to antibiotics. Her work focuses on bacterial cell-membrane proteins called efflux pumps (categorized into six families), which can pump out molecules, such as antibiotics, from the cell. The work she presented looks at intracellular accumulation of antibiotics, how efflux pumps are regulated and how these pumps assemble, highlighting recent progress in understanding the diverse nature of bacterial efflux pumps and their central role in antibiotic resistance. Jessica also discussed findings and insights that may inspire future research efforts aimed at controlling AMR. Moving forward, Jessica is exploring how antibiotics penetrate Gram-negative bacteria, such as *Escherichia coli* and *Salmonella enterica* subspecies serovar Typhimurium, with a view to developing more-effective treatments by understanding antibiotic uptake.

**Maximiliano Gutierrez** (The Francis Crick Institute, London, UK) is studying tuberculosis (TB), an infection caused by *Mycobacterium tuberculosis.* TB is a major global health problem and leading cause of death worldwide. Treatment requires at least 6 months of multidrug therapy. Max presented his latest work using high-resolution microscopy techniques to study the distribution of antibiotics in infected macrophages and how different microenvironments within macrophages affect antibiotic activity. His work revealed that localization and microenvironments control antibiotic efficacy. Max is also investigating how *M. tuberculosis* can evade host defence mechanisms and survive inside human cells. He highlighted bacterial features that promote evasion and survival, as well as the role of host immune components (including autophagy) in determining infection outcome. Given the increasing importance of multidrug resistance in the fight against TB, Max championed for greater synergy between laboratory and clinical research. He identified knowledge gaps, opportunities and challenges for integrating laboratory and clinical research to help shape strategies for TB control.

## Detection of AMR, persistence in the environment, clinical case studies, impact and innovation

**Iruka N. Okeke** (University of Ibadan, Nigeria) combines microbiology and genomics to study the mechanisms that pathogenic *E. coli*, a poorly studied causative agent of disease in children, use to cause disease and acquire AMR. Iruka presented whole-genome sequencing data to identify *E. coli* determinants underlying disease and to suggest which bacterial lineages should be prioritized for vaccine development. Iruka is well known for promoting genomic surveillance of AMR in Africa, and advocates for improving access to health care, preventive tools and antibiotics as a step toward a solution. Efficient diagnosis of infection is required to support effective use of antibiotics. Iruka discussed how basic science is essential to develop new diagnostic tools that can be scaled efficiently at a low cost, alongside pipelines to evaluate new drug targets across different resource-level settings.

**Susanna Dunachie** (University of Oxford, UK) is internationally recognized for her expertise in microbiology and immunology to progress the treatment of bacterial infection and vaccine discovery. She researches a variety of human diseases, including melioidosis, scrub typhus, TB and COVID-19. Susanna is also working with collaborators worldwide to develop laboratory research capacity, for example, working with clinical microbiologists in Southeast Asia. During her talk, Susanna reminded us that vaccines remain a fundamental public health intervention to prevent and control infection; yet, it is possible to use existing vaccines better against bacteria showing AMR and against viruses to reduce the impact of inappropriate antibiotic prescribing. We also need new and improved vaccines to prevent infection from major pathogens that are gaining AMR, such as *M. tuberculosis*. To drive this, Susanna discussed the need for a deeper understanding of human immunity to a wide variety of bacteria in health and disease, and shared data demonstrating the role of T cells in the immune response to *E. coli* and *Klebsiella pneumoniae*.

## Exploring opportunities and how DMM can help

Infection remains a primary cause of death globally and AMR is a growing problem; the development of new diagnostics, drugs and vaccines to combat AMR, therefore, requires more immediate attention. In addition, public engagement around AMR is important to understand different viewpoints, voice concerns and curb behaviours that promote AMR. More widely, AMR is a global problem that requires international cooperation to fully address and to ensure that concerns translate into funding investment for the discovery, manufacture and application of new solutions. The dangers of AMR to human health are components of a wider ‘One Health ecosystem’ that also includes animal and environmental health. Communication and cooperation across these different research communities will be required for a sustainable approach to AMR.

DMM is dedicated to advancing the development and implementation of scientific information to help address the threat of AMR. The journal's primary mission is to serve as a dissemination platform of high-quality research discovery and translational impact. Moving forward, the journal aims to promote interdisciplinary collaboration in the field of AMR by offering a platform where researchers, clinicians and key stakeholders assemble to share insights that will deliver real-world consequence.

## References

[DMM052789C1] Mostowy, S. (2022). Louis Pasteur continues to shape the future of microbiology. *Dis. Model. Mech.* 15, dmm050011. 10.1242/dmm.050011 36504391 PMC10655809

[DMM052789C2] Sanyal, S. (2023). Crossroads in virology: current challenges and future perspectives in the age of emerging viruses. *Dis. Model. Mech.* 16, dmm050476. 10.1242/dmm.050476 37728628 PMC10538290

